# E-cigarette use and respiratory symptoms in adults: A systematic review and meta-analysis

**DOI:** 10.18332/tid/174660

**Published:** 2023-12-14

**Authors:** Mohammed M. Alqahtani, Faraj K. Alenezi, Mohammed A. Almeshari, Abdullah M. Alanazi, Ziyad Ben Taleb, Mohammad E. Ebrahimi Kalan, Mary P. Martinasek, Rheese J. McNab, Rachel Culbreth, Mansour Alotaibi, Hassan Aljohani, Lynda T. Goodfellow, Taha T. Ismaeil, Saleh S. Algarni, Tareq F. Alotaibi, Mobarak K. Alqahtani, Hamoud Al-Ajel, Khalid S. Alwadeai, Nafea S. Almutairi, Eric Ford

**Affiliations:** 1 Department of Respiratory Therapy, College of Applied Medical Sciences, King Saud bin Abdulaziz University for Health Sciences, Riyadh, Saudi Arabia; 2 King Abdullah International Medical Research Center, Riyadh, Saudi Arabia; 3 Population Science, American Cancer Society, Atlanta, United States; 4 Department of Anaesthesia Technology, College of Applied Medical Sciences, King Saud Bin Abdul-Aziz University for Health Sciences, Riyadh, Saudi Arabia; 5 Birmingham Acute Care Group, Institute of Inflammation and Ageing, College of Medical and Dental Sciences, University of Birmingham, Birmingham, United Kingdom; 6 Department of Rehabilitation Health Sciences, College of Applied Medical Sciences, King Saud University, Riyadh, Saudi Arabia; 7 Public Health Program, Department of Kinesiology, College of Nursing and Health Innovation, University of Texas at Arlington, Arlington, United States; 8 School of Health Professions, Eastern Virginia Medical School, Norfolk, United States; 9 Department of Health Sciences and Human Performance, University of Tampa, Tampa, United States; 10 Department of Respiratory Therapy, Georgia State University, Atlanta, United States; 11 Department of Physical Therapy, Northern Border University, Arar, Saudi Arabia; 12 Lewis College of Nursing and Health Professions, Georgia State University, Atlanta, United States; 13 Department of Community Health Sciences, College of Applied Medical Sciences, King Saud University, AlRiyadh, Saudi Arabia; 14 Department of Behavioral and Community Health, School of Public Health, University of Maryland, College Park, United States; 15 Department of Basic Sciences, College of Science and Health Professions, King Saud bin Abdulaziz University for Health Sciences, Riyadh, Saudi Arabia; 16 Department of Health Care Organization and Policy, University of Alabama at Birmingham, Birmingham, United States

**Keywords:** e-cigarette, respiratory symptoms, systematic review

## Abstract

**INTRODUCTION:**

Electronic cigarette (e-cigarette) use is gaining popularity among adults. Monitoring e-cigarette-induced respiratory symptoms is crucial for both clinical and regulatory purposes. We systematically reviewed the current literature to understand the prevalence of respiratory symptoms among exclusive e-cigarette users, dual users, and former smokers.

**METHODS:**

Databases searched included PubMed, CINAHL, Cochrane Library, Embase, and Scopus. We included all English-language, empirical quantitative articles that explored the prevalence of e-cigarette-related respiratory symptoms. Random-effects models were utilized in conducting the meta-analyses. The quality of identified studies was evaluated using the NIH Study Quality Assessment Tools. This study is registered with PROSPERO(#CRD42020165973).

**RESULTS:**

The literature search identified 1240 references. After removing duplicates and screening for eligibility, 168 studies were included in the final review. The majority of included studies reported a wide range of adverse respiratory symptoms. The respiratory symptoms were prevalent among the exclusive e-cigarette users, dual users, and those who switched from combustible cigarettes to e-cigarettes. Further, out of the RCT studies, 5 were rated as good quality, while 3 were rated as fair. Among the observational studies, 24 were rated as good quality, and 9 were rated as fair. The two experimental studies were both rated as fair quality.

**CONCLUSIONS:**

Continued monitoring of respiratory symptoms among e-cigarette users is warranted. Due to the heterogeneity and inconsistencies among studies, which limit result interpretation and highlight the need for studies assessing causal inference, further research using robust study designs is essential. This will provide clinicians with comprehensive knowledge about the potential respiratory risks of e-cigarette use.

## INTRODUCTION

It is estimated that there were 5.66 million adults in the US who were currently (some days or every day) using e-cigarettes in 2019^1^. Among current e-cigarette users, more than 2.21 million were current cigarette smokers, more than 2.14 million were former smokers, and more than 1.30 million were never smokers^1^.

The impact of long-term dual use poses a public health concern, given the accumulating evidence regarding the detrimental health effects associated with the use of e-cigarette and traditional cigarette types^2,3^. Accordingly, there is a pressing need for a more comprehensive understanding of the broad health effects associated with e-cigarettes and the dual use of e-cigarettes and cigarettes, as the potential harms have not been comprehensively reported^4^.

Research on the health effects of e-cigarette use has been increasing and systematic reviews summarized studies on toxic constituents. For example, recent systematic reviews focused on toxic constituents in e-cigarette aerosols^5-7^, carcinogen biomarkers and the association with bladder cancer^8^, oral health^9,10^, the impact of e-cigarettes on pregnancy^11^, and cardiovascular health^12^. While several studies have examined the pulmonary effects of e-cigarettes^13,14^, this evidence has not been systematically summarized and there is a lack of knowledge regarding the potential impact of e-cigarette on pulmonary health. Specifically, the effects of e-cigarettes and dual usage of e-cigarettes and traditional cigarettes on pulmonary health and respiratory symptoms merit further research and systematic review, given the fast evolution of e-cigarettes^15^.

Some evidence suggests an association between e-cigarette use and pulmonary illness^16^. Mechanisms of lung injury following e-cigarette usage are being studied with greater frequency. One study found numerous nanoparticles and oxidants present in e-cigarette aerosols, which in turn cause mitochondrial stress, DNA fragmentation, and inflammatory stress on lung cells^17^. A recent US Centers for Disease Control and Prevention (CDC) report indicated that aerosols from e-cigarettes may also contain lead, carcinogens, and volatile organic compounds^18^. These compounds can potentially damage lung and neurocognitive development in humans, which is already proven in animal studies^19^. Using e-cigarettes has also been known to worsen asthma symptoms, which is concerning, in particular for youth, due to a recent study showing that 22.5% of asthmatic adults aged ≥18 years reported currently using e-cigarettes^20^.

In this study, we conducted a systematic review of the current empirical literature on respiratory symptoms in three groups: exclusive e-cigarette users, dual users of e-cigarettes and traditional cigarettes, and those who have switched from traditional cigarettes to e-cigarettes. This research provides valuable insights into the effects of respiratory symptoms, which could serve as a guiding resource for respiratory therapists, pulmonologists, and other healthcare professionals.

## METHODS

A systematic review of the empirical literature was conducted by following the Preferred Reporting Items for Systematic Reviews and Meta-Analyses (PRISMA) protocol (Supplementary file) . We have assessed the prevalence of respiratory symptoms among three groups; these groups included exclusive e-cigarette users, dual users (both e-cigarette and traditional cigarette users), and former smokers transitioning to e-cigarettes in an attempt to quit smoking. Therefore, we conducted a meta-analysis to examine the differences in respiratory symptom incidences among these three groups. The protocol for this study was registered with PROSPERO (#CRD42020165973).

### Data sources

Relevant publications were located through a literature search from 24 September 2021, and again on 19 April 2023. To address the research question of respiratory symptoms in e-cigarette users, a combination of database-specific subject headings and keywords were used as search strategies (Supplementary file), covering the concepts of respiratory symptoms and e-cigarettes in PubMed, Embase, CINAHL, CENTRAL, and Scopus. All articles in the English language were included with no date limits (Supplementary File).

### Study selection

Two reviewers (MA and EF) individually screened all titles and abstracts based on inclusion and exclusion criteria. Next, they independently reviewed the full text of all articles that passed the initial review, and conflicts were resolved by discussion with a third reviewer (FA).

***Inclusion criteria***

In this research review, the focus was primarily on observational studies, which include cohort, case-control, and cross-sectional methodologies, as well as intervention studies, encompassing both randomized controlled and experimental designs. The participants under consideration were adults aged ≥18 years, specifically those who reported either current or past use of e-cigarettes. Furthermore, only those studies that reported explicitly on respiratory symptoms were considered. These symptoms are defined as breathlessness, dyspnea, breathing difficulties, wheeze, cough, sputum, and phlegm. To assist in understanding the specifics of the studies included, they were categorized using the PICO criteria. These criteria delineate the population in question, the intervention or comparative component of the study, and the outcome of interest, which in this context relates to respiratory symptoms.

***Exclusion criteria***

This research review excluded several types of publications and studies. Specifically, book chapters, published systematic reviews (although their reference lists were screened for potential inclusions), non-English manuscripts, and conference abstracts lacking full-text availability, were excluded. Additionally, studies that failed to report e-cigarette use status and only reported on combusted cigarette smoking or other tobacco products like hookah, cigarette, cigarillos, chewing tobacco, snuff, snus, or dissolvable tobacco products, were not considered. Similarly, research not indicating any of the respiratory symptoms or those conducted using animal samples were also excluded from the review.

### Data extraction and quality assessment

For each included study, two reviewers independently extracted data on all outcomes. They also extracted data on the manuscript’s research design (interventional, cross-sectional, observational, or experimental), study population, participant age, and e-cigarette/traditional cigarette use. The risk of bias (ROB) in individual studies was assessed independently by two reviewers at both study and outcome levels using NIH Study Quality Assessment Tools which are guidelines by the National Institutes of Health for assessing the rigor of various research studies. They cater to different study designs, including randomized controlled trials, observational studies, and systematic reviews. These tools guide users in identifying biases and evaluating overall study quality, ensuring reliable research assessments, and rate studies as having low ROB if they had robust assessment and adjustment for study characteristics.

### Data synthesis and analysis

Random-effects models were utilized in conducting the meta-analyses. Heterogeneity among the included studies was assessed using the I^2^ statistic. All analyses were conducted using the R software.

## RESULTS

### Study characteristics

Of the 168 full-text articles assessed for eligibility, 43 studies met our inclusion criteria from eleven different countries (Supplementary file). Among these, 19 studies were conducted in the United States^21-39^, seven in the United Kingdom^40-46^ and four in Italy^47-50^. Other countries included Poland^51^, Greece^52,53^, Malaysia^54,55^, Australia^56^, Canada^57^, Saudi Arabia^58^ and Indonesia^59^. One multi-country study included a survey of respondents from France, Canada, Belgium, and Switzerland^60^. Two studies used data sources such as a worldwide survey and Internet forums to recruit e-cigarette users^61,62^.

The age groups for these studies ranged from young adults (18 years of age) to older adults (65 years). Of the 43 studies reviewed for study design, 8 were randomized controlled trials (RCTs)^25,31,40,42-45,47^, 19 were cross-sectional studies^22,24,29,30,35,37-39,41,51-53,55,56,58-61,63^, and two were experimental studies^27,28^. Two studies used an integrated, mixed-method participatory approach called concept mapping^36,62^ and another three studies used a retrospective study design^23,33,57^. The remaining nine studies are categorized as longitudinal cohort studies^21,26,34,48,50,54,64-66^ (Supplementary file).

Of the 43 studies that were reviewed, 14 presented data on studies with less than 100 subjects^22,25,27,28,31,34,45,48-50,52,59,60,62^ while eleven studies presented data with sample sizes that ranged from 100 to just under 1000 subjects ^39,40,42-44,47,53,54,56,58,66^. Finally, 15 studies gathered data from larger sample sizes of over 1000 subjects using different data sources such as surveys and online forum^21,24,26,30,35-38,41,51,55,57,61,63,64^.

***Quality assessment and risk of bias***

As the studies were cohort, cross-sectional, and randomized controlled trials, all included articles were evaluated using the NIH quality assessment tools. Five studies out of the RCT studies were rated as good quality, whereas three were rated as fair. Twenty-four observational studies were rated as good quality, nine as fair, and finally, the two experimental studies were rated as fair (Supplementary file Figure 1).

### Meta-analysis of respiratory symptoms among different e-cigarette users: Exclusive e-cigarette users, dual users and former smokers transitioning to e-cigarettes

***Any reported respiratory symptoms***

**Figure 1:**
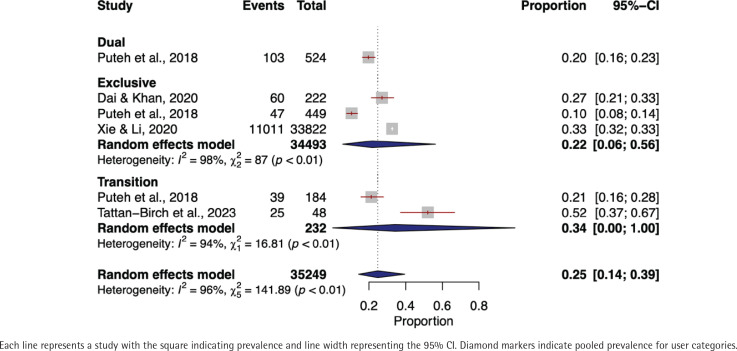
Forest plot of respiratory symptom prevalence among exclusive, dual, and transitioning e-cigarette users

In the meta-analysis evaluating the prevalence of any respiratory symptoms among exclusive e-cigarette users, a total of 34493 individuals from three distinct studies^26,38,55^ were included. Utilizing a random-effects model, we determined the pooled prevalence of any respiratory symptoms to be 22% (95% CI: 0.06–0.56). High heterogeneity was observed across the studies (I^2^=98%, p<0.01) (Figure 1).

No meta-analysis was conducted on dual e-cigarette users as there was only one study^55^ that reported it. A total of 524 individuals were included in that study and the prevalence of any respiratory symptoms was 20%.

Two studies^43,55^ involving 232 transitioning e-cigarette users were included in the meta-analysis of any respiratory symptoms prevalence, resulting in a pooled prevalence of 34% (95% CI: 0.00–1.00). A high level of heterogeneity was observed among the studies (I^2^=94%, p<0.01) (Figure 1).

***Cough***

**Figure 2:**
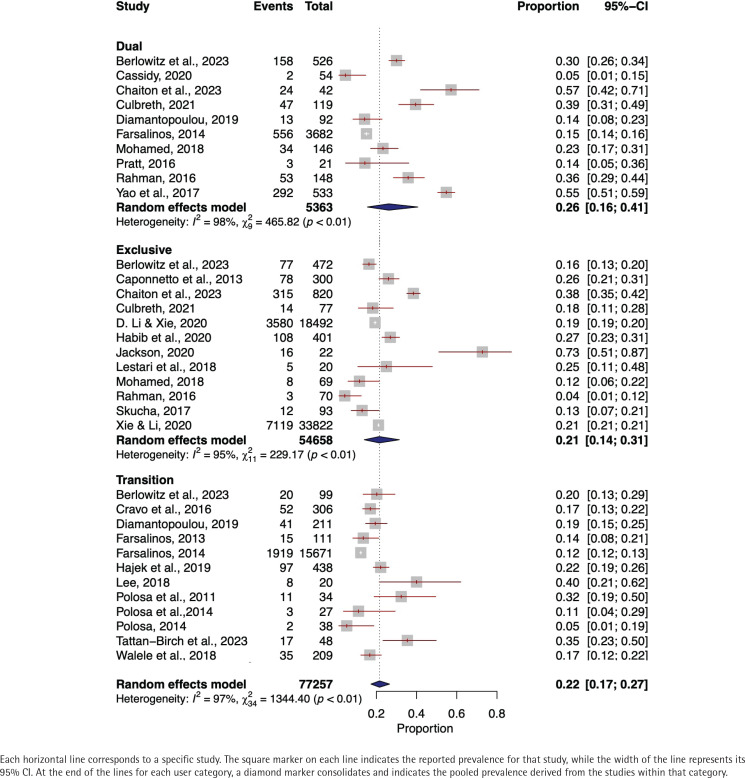
Forest plot of cough prevalence among exclusive, dual, and transitioning e-cigarette user

In the meta-analysis of cough prevalence among dual e-cigarette users, data from 5363 individuals across 10 studies were included 21,22,24,34,39,52, 54,57,61,66. A random-effects model resulted in a pooled cough prevalence of 26% (95% CI: 0.16–0.41). A high level of heterogeneity was observed among the studies (I^2^=98%, p<0.01) (Figure 2).

The prevalence of cough among exclusive e-cigarette users encompassed a total of 54658 individuals from 12 separate studies^21,24,29,38,47,51,54,57-59,63,66^. A random-effects model was employed, revealing a pooled prevalence of cough at 21% (95% CI: 0.14–0.31). A significant heterogeneity was identified across the studies (I^2^=95%, p<0.001) (Figure 2).

The cough prevalence, which encompassed 13 studies^21,31,40,42-45,48,50,52,53,61,65^ and a total of 17236 transitioning e-cigarette users, utilized a random-effects model to determine a pooled cough prevalence of 18% (95% CI: 0.14–0.23). A high level of heterogeneity was observed among the studies (I^2^=88%, p<0.01) (Figure 2).

***Phlegm***

**Figure 3:**
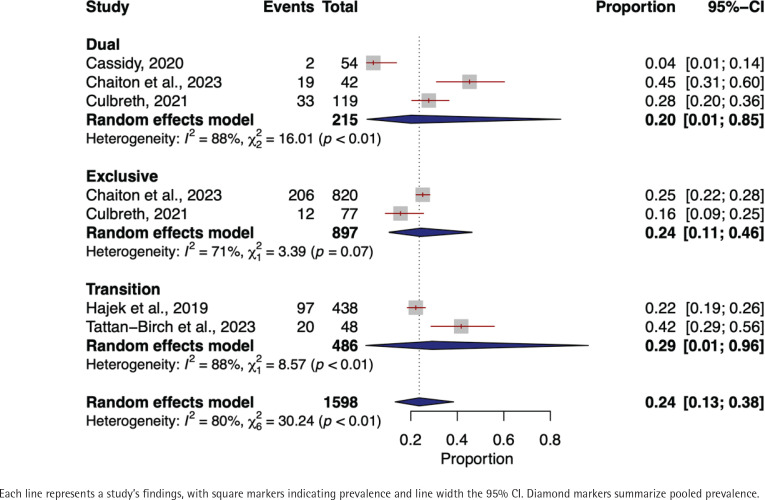
Forest plot of phlegm prevalence among exclusive, dual, and transitioning e-cigarette users

In the meta-analysis focusing on the prevalence of phlegm among exclusive e-cigarette users, we included a total of 897 individuals from two distinct studies^24,57^. The pooled prevalence of phlegm was found to be 24% (95% CI: 0.11–0.46). We noted a moderate degree of heterogeneity across the studies (I^2^=71%, p=0.07) (Figure 3).

The prevalence of phlegm among dual e-cigarette users included 215 individuals’ data from three studies^22,24,57^. The random-effects model yielded a pooled phlegm prevalence of 20% (95% CI: 0.01–0.85). The heterogeneity among studies was substantial (I^2^=88%, p<0.01) (Figure 3).

In the transitioning e-cigarette users, data from two studies^42,43^ involving 486 yielded a pooled prevalence of 29% (95% CI: 0.01–0.96). High heterogeneity across the studies was observed (I^2^=88%, p<0.01) (Figure 3).

***Shortness of breath***

**Figure 4:**
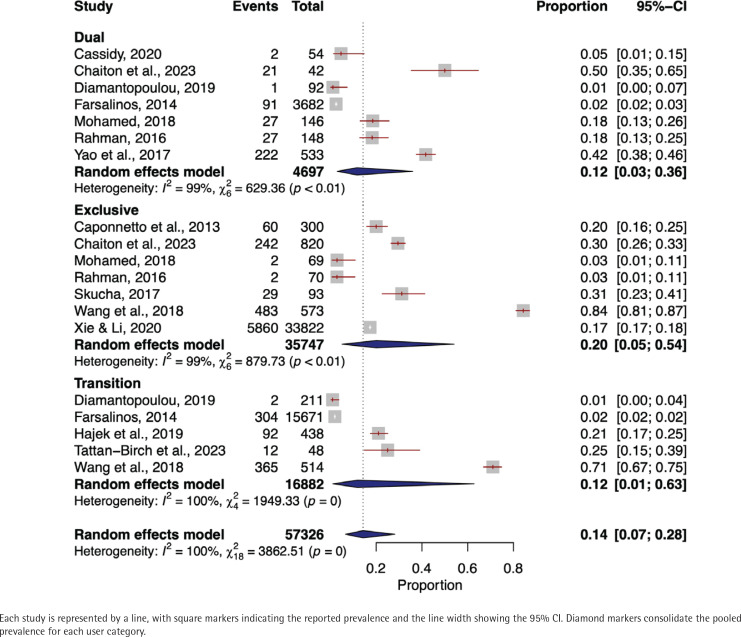
Forest plot of shortness of breath prevalence among dual, exclusive, and transitioning e-cigarette users

In the meta-analysis investigating the prevalence of shortness of breath among dual e-cigarette users included 4697 individuals from seven studies^22,39,52,54,57,61,66^. The pooled prevalence of shortness of breath was 12% (95% CI: 0.03–0.36) using a random-effects model. A high heterogeneity was noted (I^2^=99%, p<0.01) (Figure 4).

In exclusive e-cigarette users, data from 35747 individuals across seven individual studies were incorporated37,38,47,51,54,57,66. The pooled prevalence of shortness of breath was found to be 20% (95% CI: 0.05–0.54). The studies demonstrated a high degree of heterogeneity (I^2^=99%, p<0.01).

In the transitioning e-cigarette users, the shortness of breath prevalence meta-analysis encompassed five studies^37,42,43,52,61^ and 16882 individuals. The pooled prevalence was 12% (95% CI: 0.01–0.63) and high level of heterogeneity was found (I^2^=100%, p<0.01) (Figure 4).

***Wheezing***

The meta-analysis of wheezing prevalence among dual e-cigarette users included 1274 individuals from five studies^21,22,24,39,57^. The pooled wheezing prevalence was 21% (95% CI: 0.08–0.43) using a random-effects model. High heterogeneity was observed (I^2^=88%, p<0.01) (Supplementary file Figure 2).

Among exclusive e-cigarette users, data from 20807 individuals across six separate studies were included^21,24,26,35,57,63^. Using a random-effects model, the pooled prevalence of wheezing was 19% (95% CI: 0.12–0.30). The heterogeneity among studies was high (I^2^=93%, p<0.01).

Wheezing prevalence was analyzed from three studies^21,42,43^ which included 585 transitioning e-cigarette users. The pooled prevalence stood at 17% (95% CI: 0.11–0.24). The heterogeneity was found to be low (I^2^=0%, p=0.91) (Supplementary file Figure 2).

***Oropharyngeal symptoms***

In the meta-analysis of oropharyngeal symptom prevalence among dual e-cigarette users, data from 4521 individuals across four studies^39,41,52,61^ were included. The pooled prevalence of oropharyngeal symptoms was 3% (95% CI: 0.01–0.13) using a random-effects model. A moderate level of heterogeneity was observed (I^2^=78%, p<0.01) (Supplementary file Figure 3).

No meta-analysis of the prevalence of oropharyngeal symptoms among exclusive e-cigarette users was conducted as only one study reported it^58^. However, data from 401 individuals from the single study showed a prevalence of oropharyngeal symptoms of 7%.

The prevalence of oropharyngeal symptoms was analyzed across six studies^41,48-50,52,61^ with 17091 transitioning e-cigarette users, resulting in a pooled prevalence of 4% (95% CI: 0.01–0.15). Heterogeneity was found to be high (I^2^=91%, p<0.01) (Supplementary file Figure 3).

***Dry mouth***

The meta-analysis of dry mouth symptom prevalence among dual e-cigarette users incorporated 3976 individuals from three studies^54,61,66^. The pooled prevalence of dry mouth symptoms was 52% (95% CI: 0.27–0.76) using a random-effects model. A high heterogeneity was noted (I^2^=96%, p<0.01) (Supplementary file Figure 4).

For the prevalence of dry mouth symptoms among exclusive e-cigarette users, a total of 840 individuals were included from four studies^47,54,58,66^. The pooled prevalence, based on a random-effects model, was 37% (95% CI: 0.15–0.67). The studies displayed high heterogeneity (I^2^=95%, p<0.01) (Supplementary file Figure 4).

Four studies^48,50,61,65^, involving a total of 15770 transitioning e-cigarette users, were included in the meta-analysis of dry mouth symptoms prevalence. The pooled prevalence was 15% (95% CI: 0.03–0.50). The heterogeneity was found to be high (I^2^=87%, p<0.01) (Supplementary file Figure 4).

***Chest pain***

In the meta-analysis of chest pain symptom prevalence among dual e-cigarette users, a total of 4361 individuals were included from four studies^22,39,52,61^. The pooled prevalence of chest pain symptoms was 5% (95% CI: 0.00–0.38) using a random-effects model. The studies showed a high level of heterogeneity (I^2^=99%, p<0.01) (Supplementary file Figure 5).

The prevalence of chest pain symptoms among exclusive e-cigarette users, incorporated data from 817 individuals from three studies^26,29,37^. The random-effects model indicated a pooled prevalence of chest pain symptoms at 22% (95% CI: 0.04–0.66). High heterogeneity was observed among the studies (I^2^=95%, p<0.01) (Supplementary file Figure 5).

The meta-analysis of chest pain symptoms prevalence included five studies^37,42,52,53,61^ and 16945 transitioning e-cigarette users; the pooled prevalence was found to be 7% (95% CI: 0.01–0.32). High heterogeneity was noted (I^2^=100%, p<0.01) (Supplementary file Figure 5).

***Nasopharyngeal***

The prevalence of nasopharyngeal symptoms was analyzed from two studies^44,45^ which included 515 transitioning e-cigarette users. The meta-analysis yielding a pooled prevalence of 19% (95% CI: 0.00– 0.99). High level of heterogeneity was observed (I^2^=97%, p<0.01) (Supplementary file Figure 6). No meta-analysis of nasopharyngeal symptoms was conducted for exclusive e-cigarette users or dual users due to lack of studies reporting it.

***Throat irritation***

Lastly, the meta-analysis of throat irritation symptoms prevalence among dual e-cigarette users included 4836 individuals from seven studies34,39,41,52,54,61,66. The pooled prevalence of throat irritation symptoms was 15% (95% CI: 0.05–0.36) using a random-effects model ( Supplementary file Figure 7).

The meta-analysis on the prevalence of throat irritation symptoms among exclusive e-cigarette users incorporated data of 459 individuals from four studies^47,54,59,66^. Utilizing a random-effects model, the pooled prevalence of throat irritation was found to be 24% (95% CI: 0.04–0.69).

Ten studies^31,40,41,44,48,50,52,53,61,65^ involving 17737 transitioning e-cigarette users were analyzed. The pooled prevalence was found to be 17% (95% CI: 0.07–0.34). High-level heterogeneity was found (I^2^=96%, p<0.01) (Supplementary file Figure 7).

Overall, a significant difference was observed in the incidence of phlegm, throat irritation, chest pain, dry mouth, shortness of breath, oropharyngeal symptoms, wheezing, cough, and any respiratory symptoms among the three distinct groups: exclusive e-cigarette users, dual users, and former smokers transitioning to e-cigarette use.

## DISCUSSION

This review contributes to the literature and knowledge surrounding the association between e-cigarette use and the manifestation of respiratory symptoms in adults. While animal studies have highlighted the deleterious effects of e-cigarettes on the pulmonary system, there remains a gap in our understanding of the association between e-cigarette-induced respiratory symptoms and usage status in human subjects. For instance, among current dual users, it is ambiguous whether respiratory symptoms are a consequence of e-cigarette use or traditional cigarette smoking. Further, it is uncertain whether existing symptoms are remnants of past smoking habits that have persisted into current e-cigarette use among former smokers. Therefore, it is crucial to conduct studies with robust research designs to track these symptoms longitudinally to determine the temporal relationships between e-cigarette use and the incidence of respiratory symptoms among different e-cigarette users.

Overall, our review found that e-cigarette users reported presence of respiratory symptoms. In our comprehensive analysis, those who exclusively used e-cigarettes displayed significant occurrences of different respiratory issues such as coughing, phlegm production, breathing difficulties, wheezing, dry mouth, chest discomfort, and irritation in the throat. Moreover, our analysis encompassed several studies that examined respiratory symptoms in individuals who used both e-cigarettes and cigarettes; the results indicated significant instances of cough, phlegm, shortness of breath, wheezing, dry mouth, chest pain, and throat irritation. The meta-analysis revealed the following incidences of respiratory symptoms among transitioning e-cigarette users: cough, phlegm, shortness of breath, any respiratory symptoms, wheezing, oropharyngeal symptoms, dry mouth symptoms, nasopharyngeal symptoms, chest pain symptoms, and throat irritation symptoms. These data suggest a significant impact of e-cigarette usage on respiratory health, underlining the need for further investigation.

Many studies reported different respiratory symptoms, strongly suggesting the toxic effects of e-cigarette usage that span across multiple reported symptoms^10,18,67,68^. The co-occurrence of multiple symptoms across e-cigarette user categories emphasizes the potentially harmful effects of e-cigarettes. It is noteworthy that individuals who only used e-cigarettes and were not former traditional smokers (exclusive only e-cigarette users) also experienced adverse respiratory symptoms, including increased cough, dry mouth/mouth irritation, phlegm, wheezing, shortness of breath, chest pain, and palpitations^20,69^. As demonstrated previously by animal models, e-cigarette exposure is linked to an increase in oxidative stress and inflammatory cytokines in bronchoalveolar lavage samples, increased mucus production, and impaired pulmonary immunological function^70^. Thus, it is not surprising that human self-reported respiratory symptoms often include multiple, overlapping symptoms from e-cigarettes. Future longitudinal studies need to examine the development of symptoms across the life course of e-cigarette users, particularly since these devices have only emerged in recent years and diversifying at a rapid pace.

Reported respiratory symptoms among dual users is concerning. In this review, our meta-analysis encompassed multiple studies examining respiratory symptoms among dual e-cigarette users, revealing significant incidences of cough, phlegm, shortness of breath, and wheezing. These results, along with data on other symptoms such as dry mouth, chest pain, and throat irritation, suggest a notable impact of dual e-cigarette usage on respiratory health, which could also be an effect of the high nicotine levels from using both traditional cigarettes and e-cigarettes simultaneously^22,24,37,39,41,52,54,61,64,66^. Although it is hard to conclude the origin of the respiratory symptoms among dual users, it is known that e-cigarettes have an abundance of other respiratory system irritants such as carbonyls (e.g. aldehydes), volatile organic compounds (e.g. acrolein)^71^. Because of the potential synergistic effects of e-cigarette and traditional cigarette dual usage, cessation interventions and primary prevention education are urgently needed. Thus, interventions which address the potential comorbidities and cultural considerations of dual users may be beneficial.

There is a pressing need for research to discern the long-term impact of early e-cigarette initiation on pulmonary health throughout the life course, particularly among youth who introduce e-cigarettes as their first tobacco products.

Currently, although researchers are somewhat divided regarding whether e-cigarettes can or should be used as an aid for smoking cessation, in fact, studies have shown that e-cigarettes provide mixed results in terms of smoking cessation^46,72^. While e-cigarettes were initially marketed as a smoking cessation tool, it is clear that e-cigarettes are not harmless and the uptake among current smokers often results in dual use rather than cessation of tobacco altogether^73,74^. Proper education on adequate smoking cessation tools is needed for current smokers to correct the inaccurate marketing messages of e-cigarettes as cessation tools^73,74^, which is not an approved cessation tool in the US and several other nations.

Future research should include more long-term studies of animal models, utilizing predictive modeling for respiratory symptoms of e-cigarette users across the life course. Interventions are also warranted for populations at-risk for e-cigarette use, particularly dual usage. Further, longitudinal epidemiological studies are needed to dissect the trajectory of respiratory symptoms development among e-cigarette users.

Our study may contribute to better understand the prevalence of respiratory symptoms among e-cigarette users. Given the rising popularity of e-cigarettes and their potential health implications, understanding the respiratory risks associated with their use is crucial for public health. These studies provide valuable insights into the prevalence, incidence, and impact of respiratory symptoms, helping to inform policies, interventions, and healthcare strategies aimed at mitigating potential health risks. By assessing the respiratory health of e-cigarette users, we can gain a better understanding of the potential harms and inform evidence-based approaches to protect and promote respiratory well-being in this population.

### Limitations

While this systematic review presents notable insights into self-reported respiratory symptoms linked to e-cigarette use, several limitations warrant mentioning. First is the heterogeneity of the study samples, methodologies, and outcomes across the included studies for the meta-analysis. Second, this study did not encompass adolescent populations, a demographic that could potentially exhibit different e-cigarette use patterns compared to adults, and hence may display disparities in respiratory symptom prevalence warranting future investigation. A significant number of the symptoms were subjectively reported by the individuals rather than being identified using objective or observational metrics, which introduces the potential for recall and social desirability biases. As such, these symptoms require verification in larger and multicentric longitudinal studies globally. This study did not differentiate between e-cigarettes, whether any of the included assessed modified e-cigarette cartridges and liquids and heated tobacco products in assessing their health effects, highlighting the need for future research to address this limitation and examine the specific health impacts of each product. Moreover, we have noted that individuals who use e-cigarettes may also have concurrent health conditions and a history of traditional smoking. Lastly, considering the high degree of heterogeneity observed among the studies, the majority of results were interpreted using a random-effects model. Hence, there is a need for higher-quality RCTs and prospective studies to assess causality, with a focus on exclusive e-cigarette use. Despite these limitations, this systematic review contributes to the expanding body of knowledge regarding the impact of e-cigarette use on respiratory health.

## CONCLUSIONS

In this systematic review, our meta-analyses of exclusive e-cigarette users, dual e-cigarette users, and transitioning e-cigarette users demonstrated significant incidences of various respiratory symptoms, emphasizing the impact of e-cigarette usage on respiratory health and the need for further research in this area. Effective e-cigarette cessation interventions are needed to prevent respiratory symptoms and respiratory disease, and subsequently improve health outcomes. The results from this study will inform clinical recommendations/guidelines for e-cigarette users and dual users of e-cigarettes and traditional cigarettes.

## Data Availability

The data supporting this research are available from the authors on reasonable request.
