# Supplementary material for: E-cigarette use and respiratory symptoms in adults: A systematic review and meta-analysis
Source: Tob Induc Dis. 2023 Dec 14;21:168. doi: 10.18332/tid/174660 (PMC10720266; doi:10.18332/tid/174660)
Supplement: Supplementary file 1 [file TID-21-168-s1.pdf]

Figure 1. a: Quality assessment of included studies based on the NHLBI Randomized Controlled Trials Studies Quality Assessment Tool.

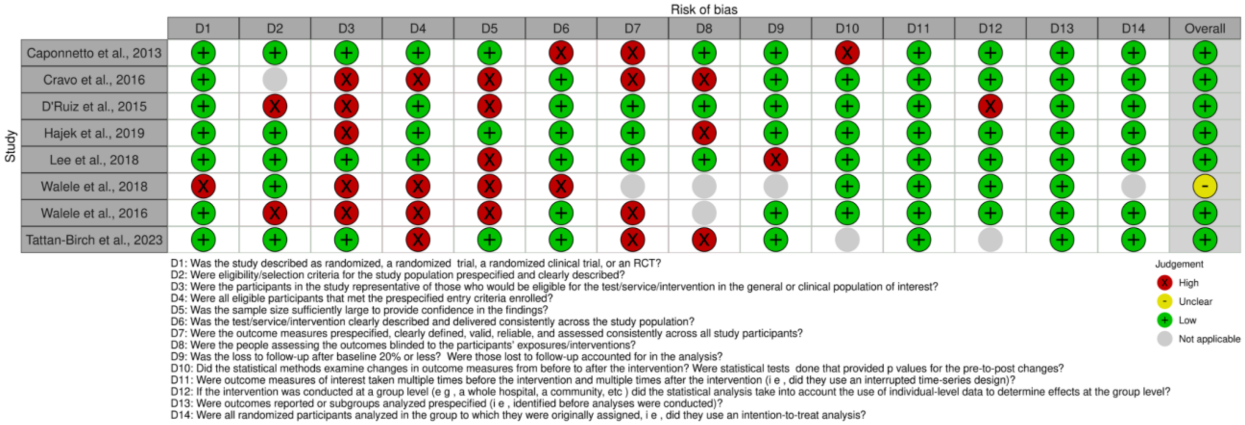

Figure 1.b: Quality assessment of included studies based on the NHLBI Observational Cohort/ Cross-Sectional Studies Quality Assessment Tool.

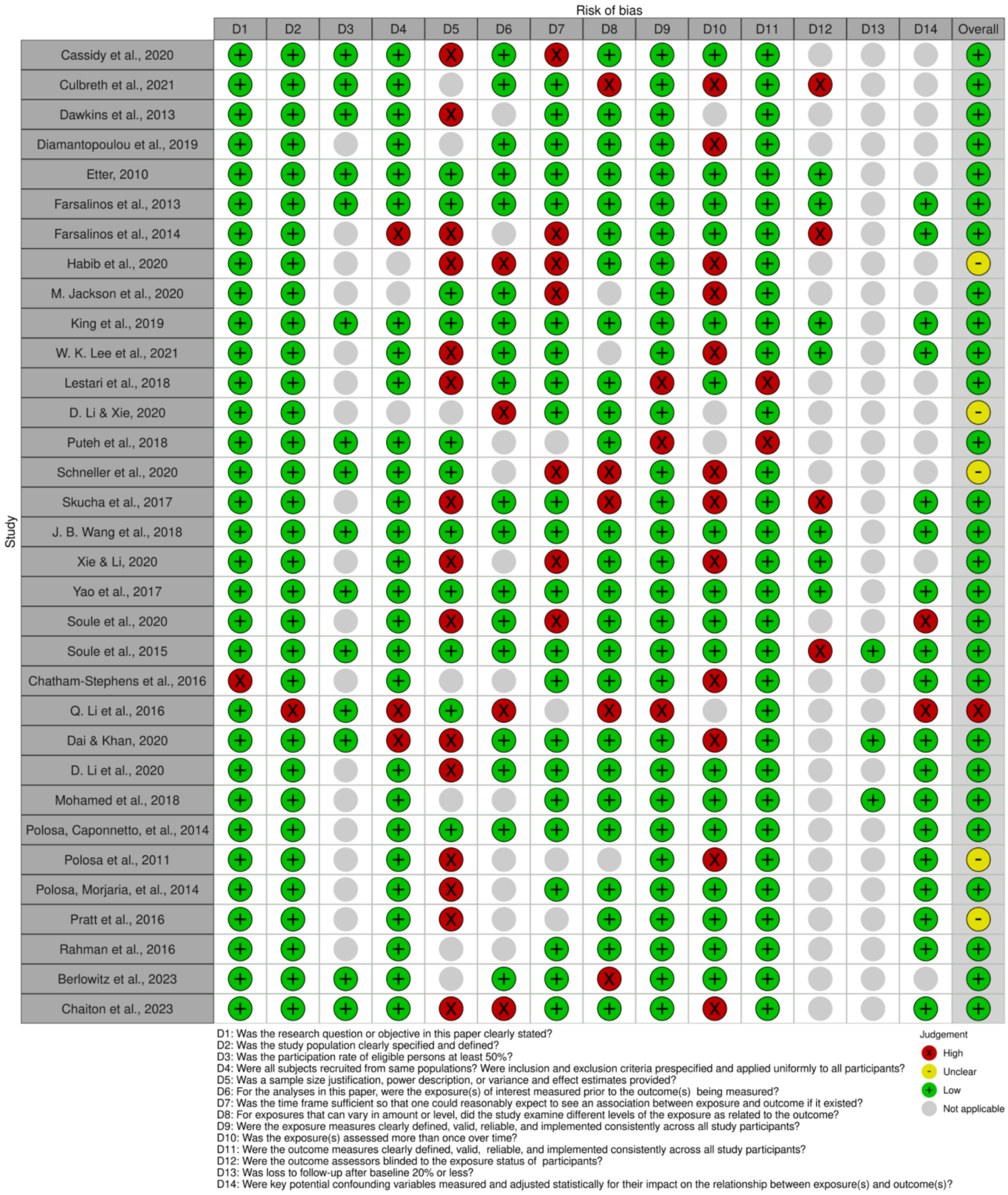

Figure 1.c: Quality assessment of included studies based on the NHLBI Experimental Studies Quality Assessment Tool.

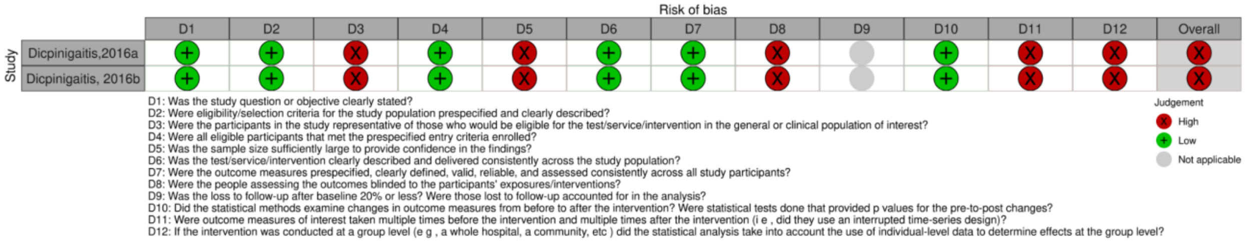

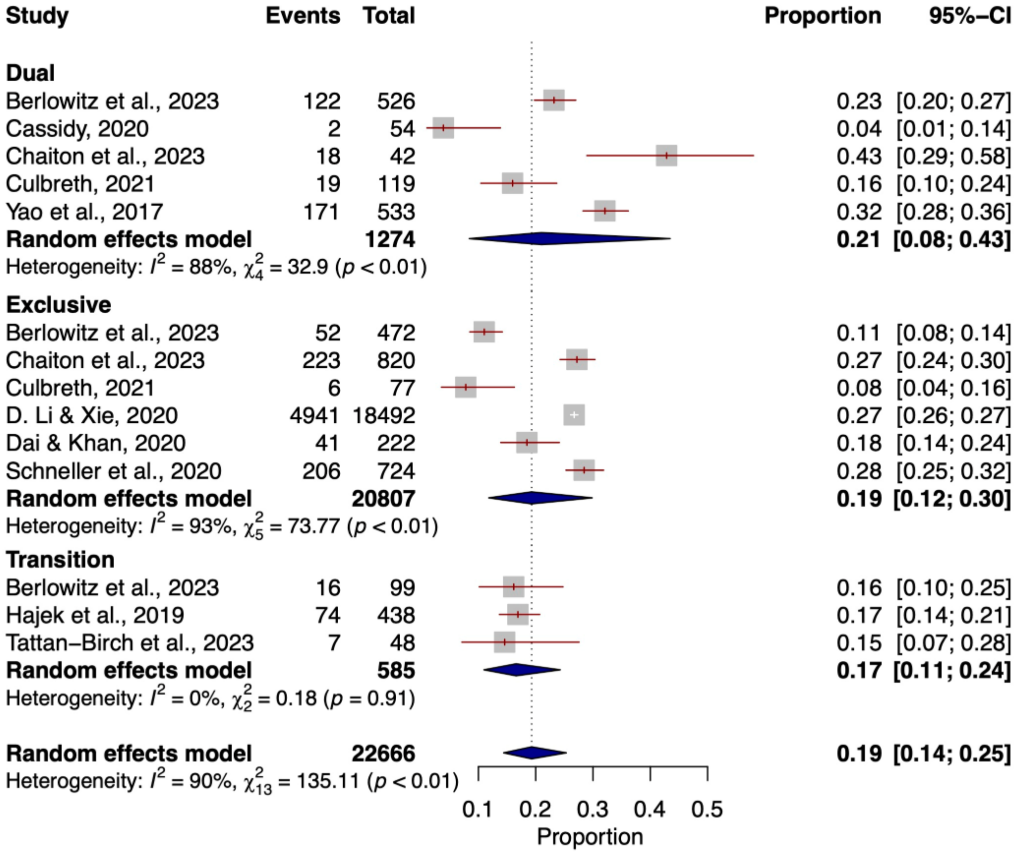

Figure 2: Forest plot of wheezing prevalence among dual, exclusive, and transitioning e-cigarette users. Legend: Forest plot depicting wheezing prevalence in distinct e-cigarette user categories. Each represented study features a line, with square markers indicating the calculated prevalence and the line width demonstrating the 95% CI. Diamond markers sum up the pooled prevalence for each user type.

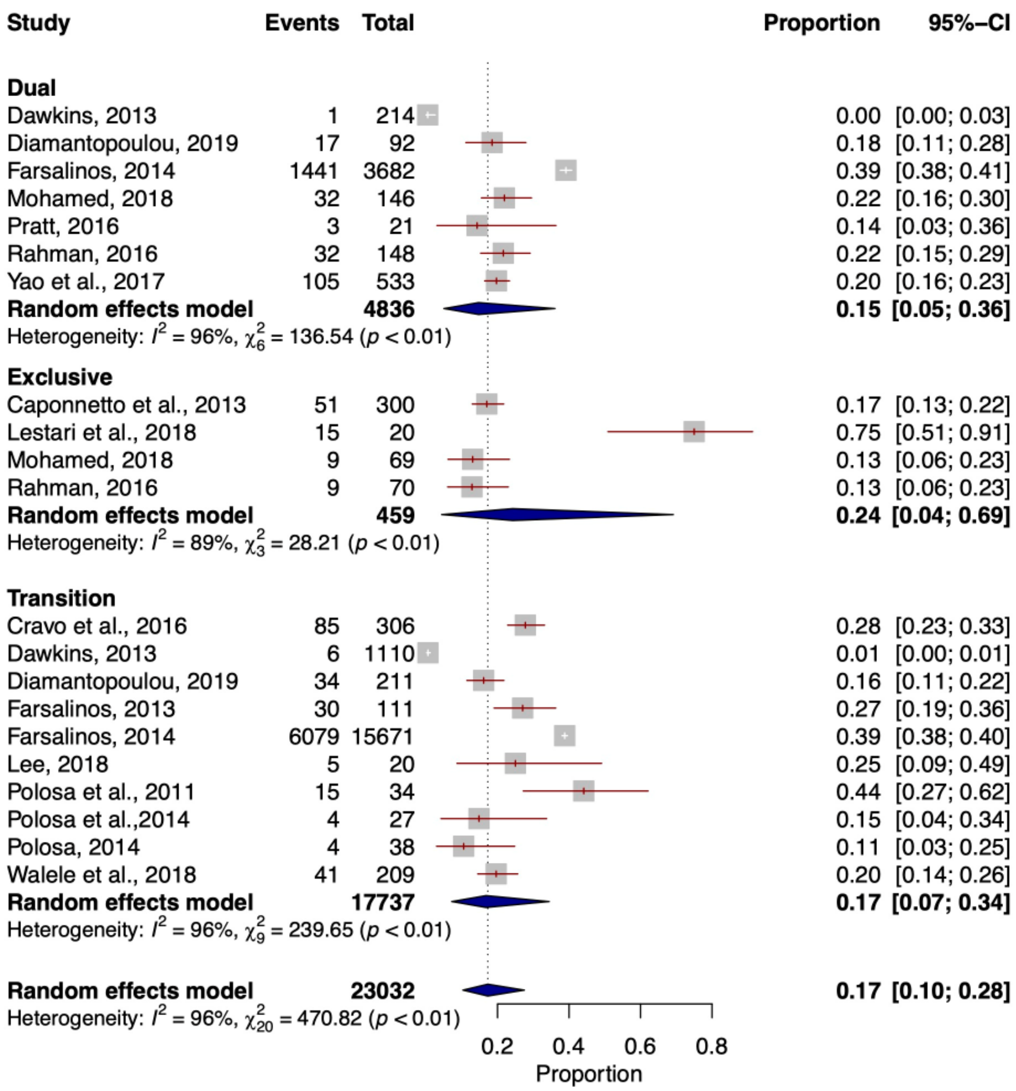

Figure 3: Forest plot of oropharyngeal symptom prevalence among dual, exclusive, and transitioning e-cigarette users. Legend: This forest plot delineates the prevalence of oropharyngeal symptoms across various e-cigarette user categories. Each study is denoted by a line, with square markers reflecting the reported prevalence and the line width displaying the 95% CI. Diamond markers aggregate the pooled prevalence for each user group.

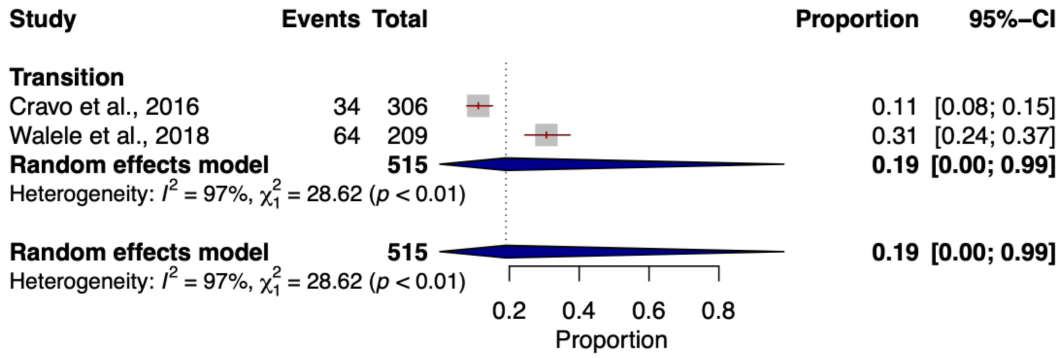

Figure 4: Forest plot of dry mouth symptom prevalence among dual, exclusive, and transitioning e-cigarette users. Legend: This forest plot highlights the prevalence of dry mouth symptoms in different e-cigarette user groups. Each study is represented by a line, with square markers showing the reported prevalence and the line width indicating the 95% CI. Diamond markers summarize the pooled prevalence for each user category.

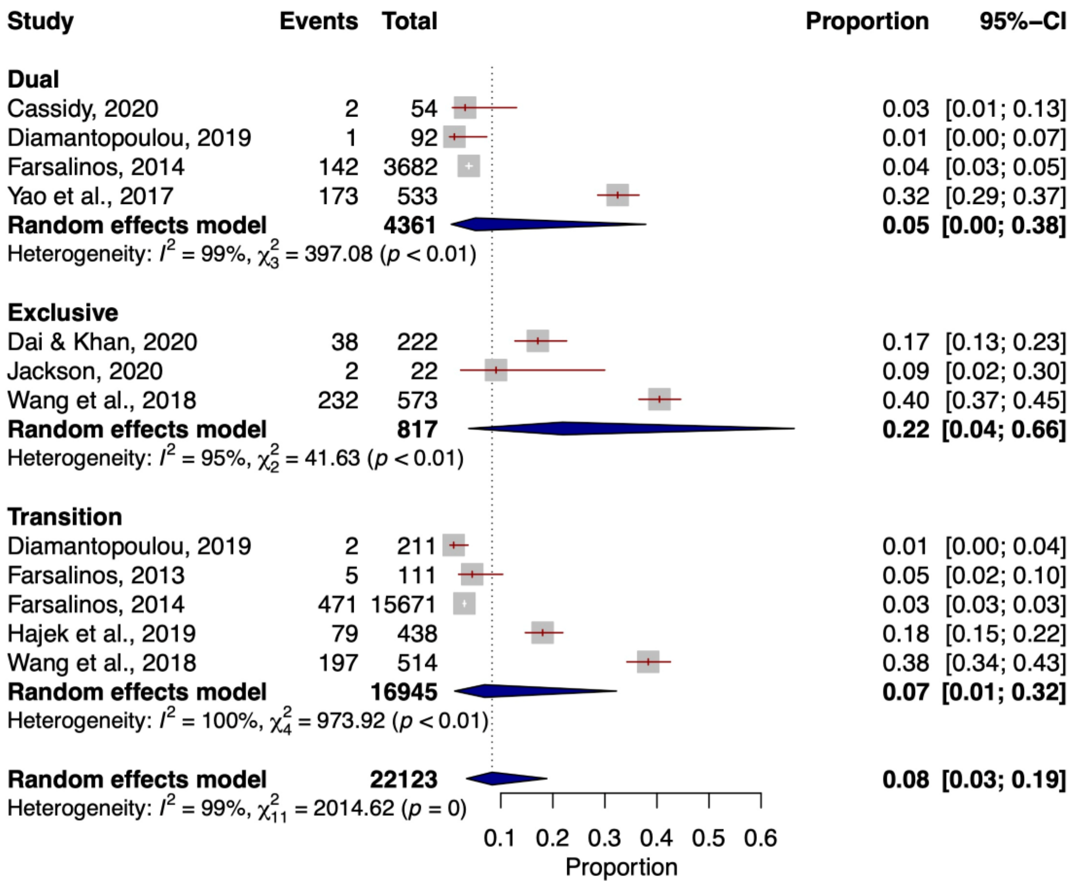

Figure 5: Forest plot of chest pain symptom prevalence among dual, exclusive, and transitioning e-cigarette users. Legend: This forest plot illustrates the prevalence of chest pain symptoms across e-cigarette user groups. Each study is denoted by a line, with square markers reflecting

the reported prevalence and the line width displaying the 95% CI. Diamond markers aggregate the pooled prevalence for each user group.

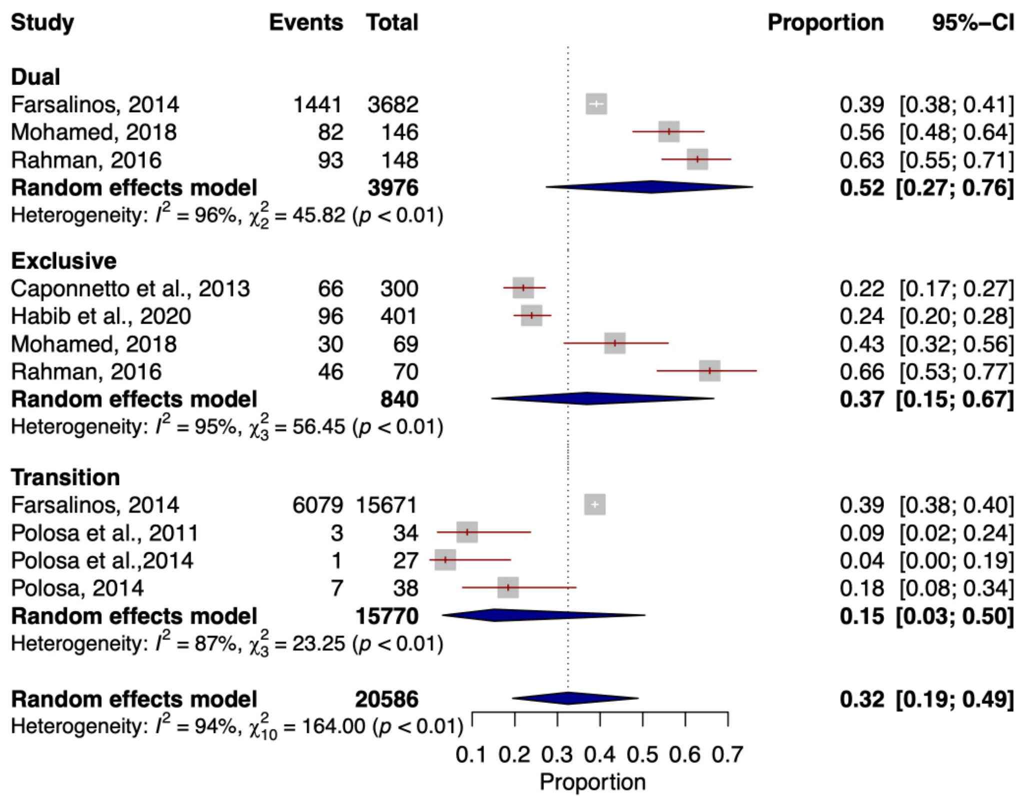

Figure 6: Forest plot of nasopharyngeal symptom prevalence among transitioning e-cigarette users. Legend: This forest plot depicts the prevalence of nasopharyngeal symptoms in transitioning e-cigarette users. Each study is represented by a line, with square markers indicating the reported prevalence and the line width showing the 95% CI. Diamond markers consolidate the pooled prevalence for the user group.

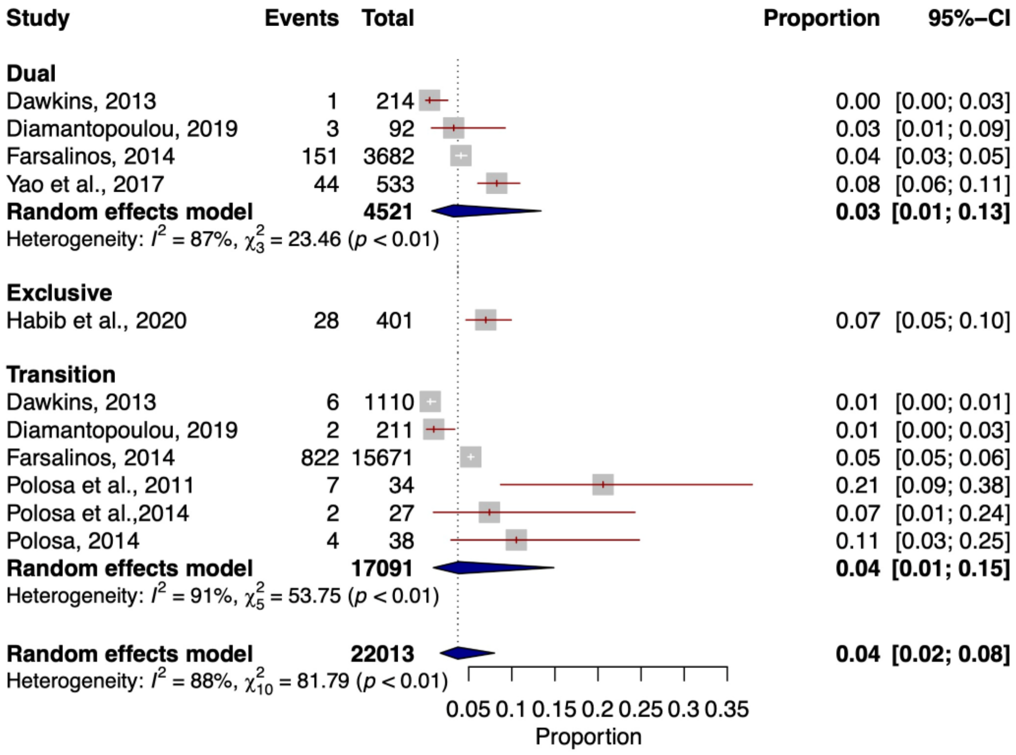

Figure 7: Forest plot of throat irritation symptom prevalence among dual, exclusive, and transitioning e-cigarette users. Legend: This forest plot showcases the prevalence of throat irritation symptoms across different e-cigarette user groups. Each study is denoted by a line, with square markers reflecting the reported prevalence and the line width indicating the 95% CI. Diamond markers summarize the pooled prevalence for each user category.

© **2023 Alqahtani M.M. et al.**
